# 

**Published:** 1854-12

**Authors:** 


### CLOSE OF THE VOLUME.

With this number the second volume of our new series is completed. It does not become us to speak of the manner in which the task has been executed; our readers have the results before them, and will judge for themselves. We wish simply here to say, that preparations have been made for continuing the Journal, and that the first number of our third volume will be issued early in January. We tender our cordial salutations to our readers, and wish them a happy New Year!

### NEW PUBLICATIONS.

A number of valuable medical works, issued within a few months past, from the publishing houses of Philadelphia and New York, are on our table, but in our present number we can do no more than merely give their titles.

Prominent among them are the beautiful volumes of *Hassall on Microscopic Anatomy*, a work of acknowledged ability and interest. It has been republished from the English edition, in befitting style, by the Messrs. Wood, of New York.

To Messrs. Lippincott, Grambo & Co., of Philadelphia, we are indebted for a kindred work—*The Manual of Human Microscopical Anatomy*, by Kölliker, one of the most zealous of all the cultivators of this modern science. As proof of the excellence of his "manual" we may mention, that it has been deemed worthy of translation by the Sydenham Society It will become a standard work on the subject of microscopical anatomy.

From Messrs. Keith & Co., of New York, we have received a beautifully executed work, entitled *Positive Medical Agents;* being a treatise on the new alkaline, resinoid, and concentrated preparations of indigenous and foreign medical plants. The work is published by authority of the American Chemical Institute, and will be found a convenient and useful manual by he practising physician.

*A Manual of Pathological Anatomy*, by C. Handfield Jones, M. B., F. R. S., and Edward H. Sieveking, M. D., has been issued by Messrs. Blanchard and Lea, in their accustomed style of accuracy and neatness. The work is destined to take a high rank among the treatises on this subject. It is fresh, consise and practical. Pathological anatomy as it exists at the present day is faithfully reflected from its pages.

The same publishers have issued *West's Lectures on Ulceration of the Os Uteri;* which will be read with great attention by American physicians, with whom the subject to which this work refers is exciting unusual interest at the present time. Dr. West may be regarded as at the head of that body of practitioners who are skeptical as to the magnitude of the evils growing out of ulceration of the os uteri, and as to the value of the speculum uteri in examinations of the uterus. In our next number we shall commence the publication of a series of lectures, by Prof. Miller, in reply to the views of Dr. West on this subject.

*Physician's Visiting List for* 1855. Messrs. Lindsay & Blakiston have sent out in advance this acceptable annual. It contains, besides an almanac and table of signs, a list of poisons and their antidotes, with blank leaves for all the memoranda that the physician in his professional rounds will find it necessary to make;

The "Visiting List," we believe, is beginning to be felt as a necessity by all physicians in extensive practice.

*Wood's Catalogue.* The publishers S. S. &. W. Wood, N. York, are sending to all orders, which are post-paid, copies of the catalogue of their extensive and varied assortment of books. The physician has only to run his eye over its pages to see what new books have been published in any of the departments of medicine.

*Comstock & Comings' Physiology.* The Messrs Wood have issued this work, designed specially for schools, academies, and colleges, in a very attractive form, and it ought to become a popular work. The authors have simplified their interesting subject sufficiently for the comprehension of the youngest student of physiology, and the publishers have supplied all the illustrations requisite. Physiology ought assuredly to form a part of the curriculum of studies in every high school and college, whether for girls or boys, and we do not know a text-book better fitted for such students than this treatise of Comstock & Comings. Its quarto form favors the introduction of larger and more striking cuts than are usually found in such works.

*Bushnan's Physiology.* This is another work the object of which is to popularize science. Its author, a London physician, is a man thoroughly versed in the science of which he treats, and though his limits are narrow he has contrived, in his brief pages, to give a highly interesting and satisfactory summary of the facts and principles in physiology. Few readers would take up his work without finding in it something new, and none could peruse it without instruction. It is amply illustrated, and may be commended especially to readers who desire to see the subject exhibited in its most condensed form. It is published by Blanchard & Lea.

The above mentioned works have been forwarded to us by the publishers through E. Maxwell, Esq., at whose store they may be found.

